# Evaluating cone cut in rectangular collimation in intraoral radiography: application of ALADA and radiation stewardship

**DOI:** 10.1007/s00784-023-05158-0

**Published:** 2023-08-04

**Authors:** D. Clark-Perry, W. E. R. Berkhout, G. C. Sanderink, D. E. Slot

**Affiliations:** 1 Department of Periodontology, Academic Centre for Dentistry, Amsterdam (ACTA), University of Amsterdam and Vrije Universiteit Amsterdam, Gustav Mahlerlaan 3004, 1081 LA Amsterdam, The Netherlands; 2 Department of Oral Radiology, Academic Centre for Dentistry, Amsterdam (ACTA), University of Amsterdam and Vrije Universiteit Amsterdam, Amsterdam, The Netherlands

**Keywords:** Radiograph, Cone cut, Rectangular collimator, Diagnostics

## Abstract

**Objective:**

Rectangular collimation is a popular method used in intraoral radiography to reduce patient exposure to ionizing radiation. One of the perceived drawbacks of rectangular collimation is the possibility of an increase in cone cut errors ultimately impacting the diagnostic value of the radiographs. Thus, the aim of this study was to explore the frequency of cone cut errors in radiographs taken using a rectangular collimator.

**Materials and methods:**

Radiographs taken using PSP plates at Academic Center for Dentistry Amsterdam in the Netherlands by staff and students from January to December 2015 were assessed for cone cut errors. The radiographs were grouped as bitewings, front teeth, inferior premolars and molars, and superior premolars and molars and categorized as no cone cut, cone cut but diagnostically usable, and cone cut but diagnostically not usable. The results were entered into Microsoft Excel and analyzed thereafter.

**Results:**

A total of 53,684 radiographs were assessed, 79% had no cone cut errors and consequently 21% had some degree of cone cut. However, the diagnostic value was unaffected in 18% of the radiographs with cone cut. Only 3% of the radiographs were deemed diagnostically unusable due to cone cut. The most common area of cone cut was in the premolar and molar areas while cone cut in the front teeth was least likely to be diagnostically unusable.

**Conclusion:**

Cone cut from the use of a rectangular collimator does not seem to result in an increase of diagnostically unusable radiographs. Thus, rectangular collimation should be preferred as it decreases the amount of radiation exposure to the patient while producing diagnostically usable radiographs and thus allowing the dental professional to adhere to the ALADA principle and practice radiation stewardship.

**Clinical relevance:**

*Scientific rationale for the study*: rectangular collimation is a method used to reduce patient exposure to ionizing radiation; however, this benefit is negligible if radiographs must be retaken due to cone cut errors that make the radiograph diagnostically unusable. Therefore, the aim of this study was to explore the frequency of cone cut in radiographs taken using a rectangular collimator. *Principal findings*: cone cut was observed in 21% of the radiographs; however, only 3% of the radiographs were considered diagnostically unusable. *Practical implications*: rectangular collimation does not result in a high number of diagnostically unusable radiographs and should be used to reduce patient exposure to ionizing radiation.

## Introduction

In dentistry, radiography is used to identify oral pathosis and anomalies. Dental professionals are obligated to reduce patient exposure to ionizing radiation by adhering to the optimization principle (also known as ALARA: as low as reasonably achievable) [1]. This means that the patient’s radiation exposure should be kept to an acceptable minimum taking into account the diagnostic value of the radiograph.

Rectangular collimation results in at least 40% less radiation exposure to patients according to a systematic review that assessed all sensor modalities including F-speed film, PSP plates, and digital sensors [2]. However, the perceived disadvantage of rectangular collimation is the potential increase in errors like cone cutting. Cone cut occurs at the edge of the film and is often due to misalignment of the collimator and/or the film. When the film is incompletely covered by the radiation beam, a white surface on the radiograph appears. The occurrence of small cone cuttings does not necessarily affect the diagnostic quality. However, in some cases, if the size of the unexposed area (white area) is large, it can make the diagnostic area of the radiograph unreadable. In this situation, the radiograph should be retaken but the consequence is exposing the patient to twice as much ionizing radiation.

In 1983, Horton et al. [3] examined a total of 3801 radiographs. Half were taken using film with a rectangular collimator and the other half were taken with a round collimator. A total of 156 radiographs showing unexposed areas due to cone cut were detected and 86% appeared with rectangular collimation. This finding was confirmed in a study conducted by Parrot and Ng[4], which showed that there was an increase in the incidence of cone cut errors on film radiographs from 3.3 up to 20.9% when rectangular collimation was used instead of round collimation. Consequently, the patient still received less radiation with rectangular collimation when compared to round collimation, because of the reduced size of the exposure. Overall, the theme in the literature appears that patients receive a reduction in radiation dose when a rectangular collimator is used [5].

Students at the Academic Center for Dentistry Amsterdam (ACTA—dental school of the University of Amsterdam and VU Amsterdam) take radiographs with PSP plates using rectangular collimation. The aim of this study was to explore the frequency of cone cut in radiographs taken with PSP plates using a rectangular collimator, where the cone cut occurred most frequently, and whether the cone cut impacted the diagnostic quality of the radiograph and explore.

## Materials and methods

This study was designed as a retrospective analysis. For preparation, the Strengthening the Reporting of Observational Studies in Epidemiology guidelines for reporting observational studies (STROBE) [6] and the Reporting of studies Conducted using Observational Routinely collected Data (RECORD) [7] guidelines were followed. The Institutional Review Board of the Academic Centre for Dentistry Amsterdam approved this retrospective analysis (2021–91,286). This retrospective study included all radiographs taken by staff and students at ACTA from January to December 2015. In 2015, there were a total of 15 X-ray machines of 4 different brands, all equipped with rectangular collimators. The intraoral receptors used for every radiograph that was taken were photostimulable phosphor (PSP) plates.

Rectangular collimation was used for all radiographs taken in 2015. The radiographs were examined by two dental students (JK, ES) trained by their supervisor (GS) and confirmed by author DCP.

**Fig. 1:**
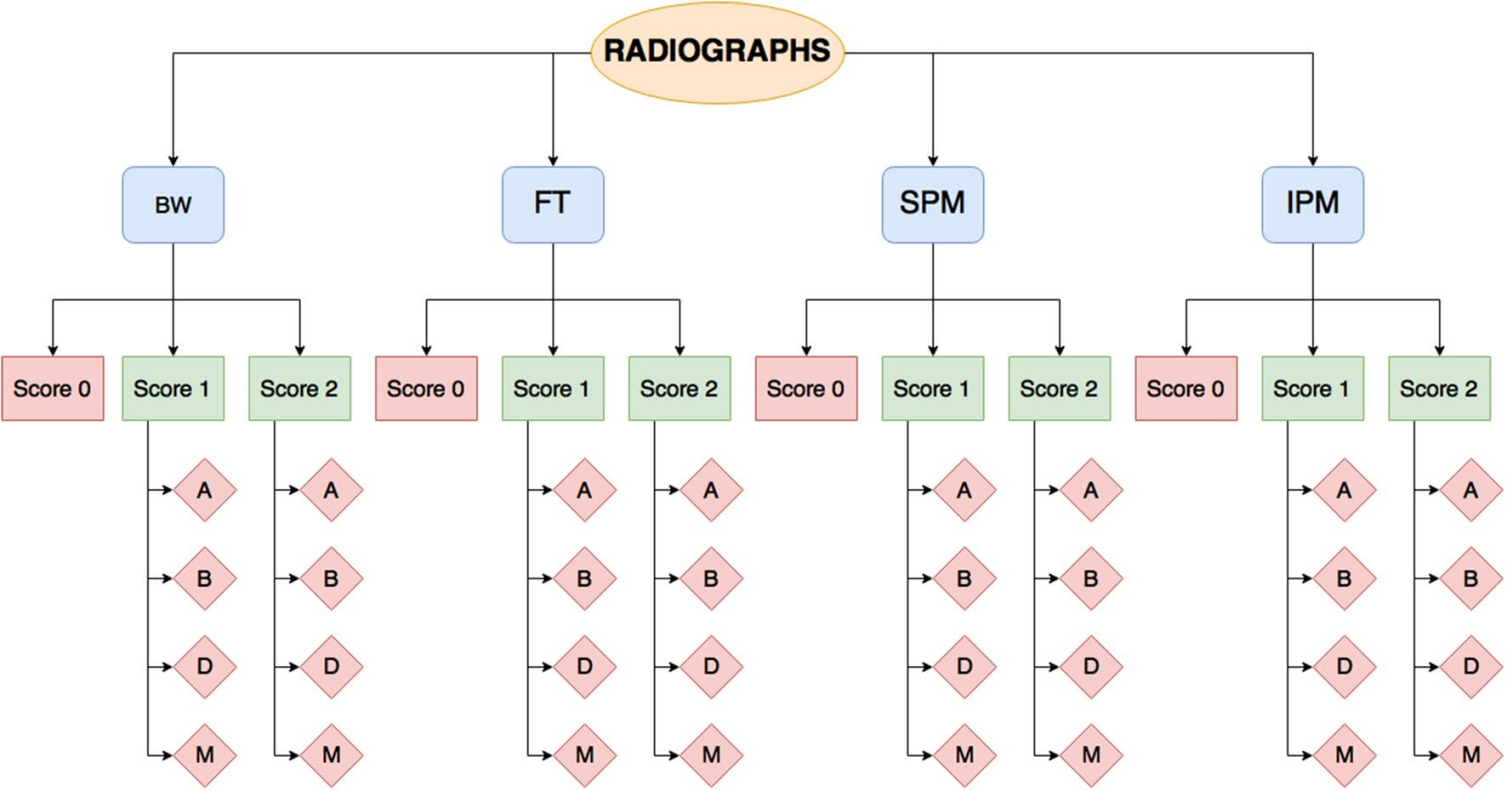
Distribution of intraoral images based on type of radiograph: bitewing (BW), front teeth (FT), superior premolar and molar (SPM), or inferior premolar and molar (IPM). The score of the radiograph: score 0: no cone cutting; score 1: cone cutting that does not affect diagnostic value; score 2: cone cutting that does affect diagnostic value. Where the cone cut occurred: above (A), below (B), mesial (M), or distal (D)

**Fig. 2:**
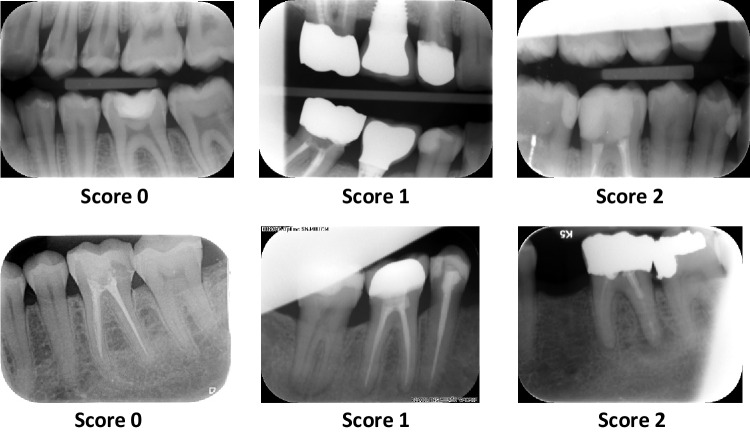
Example of scores 0, 1, and 2 on bitewing and periapical radiographs (based on score classification from Parrot and Ng [4]). Score 0: no cone cutting. Score 1: cone cutting that does not affect diagnostic value. Score 2: cone cutting that does affect diagnostic value

The radiographs were divided into 5 groups: bitewings (BW), front teeth (FT), superior premolar and molar (SPM), inferior premolar and molar (IPM), and no patient (NP). The NP group contained nonclinical images such as extracted teeth, referral patient images from external practices, or images too vague to classify. After the images were arranged as a full mouth series, the four groups were classified further into no cone cutting (score 0), cone cutting that does not affect diagnostic value (score 1), and cone cutting that does affect diagnostic value (score 2) (Fig. 1 and Fig. 2).

**Fig. 3:**
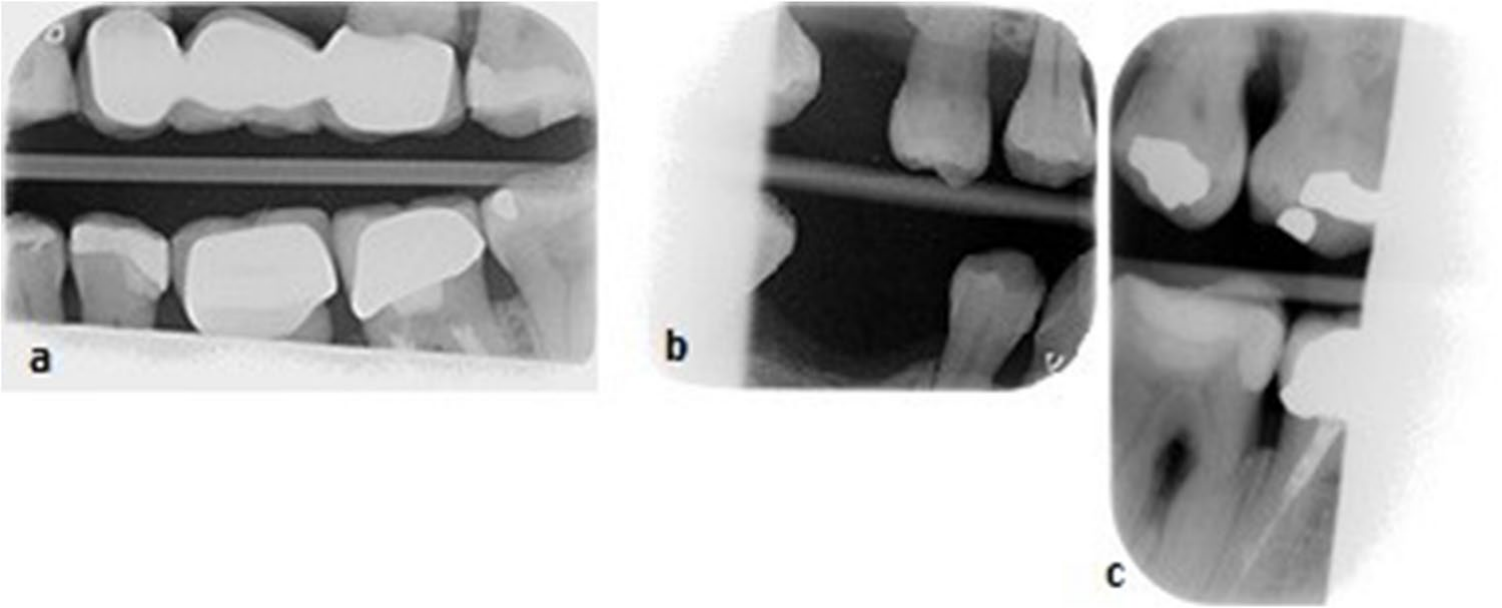
Bitewings with cone cutting that affects the diagnostic quality (score 2). The bone level next to the teeth was not visible at more than one site due to cone cutting (**a**). The number of missing teeth was more than one due to cone cutting (**b**). The number of missing contacts of approximal surfaces was more than one due to cone cutting (**c**)

The influence on the diagnostic value of the BW was based on the following criteria:

1. The bone level next to the teeth was not visible at more than one site due to cone cutting (Fig. 3a).
2. The number of missing teeth was more than one due to cone cutting (Fig. 3b).
3. The number of missing contacts of approximal surfaces was more than one due to cone cutting (Fig. 3c).

If a radiograph met one of the items above, it was classified as unusable and assigned a score of 2. Every other case of cone cutting was usable and assigned a score of 1. The classification was only based on cone cut errors. Note, when a radiograph with a cone cut was diagnostically unusable due to factors other than cone cut, such as overexposure, it was classified as usable.

**Fig. 4:**
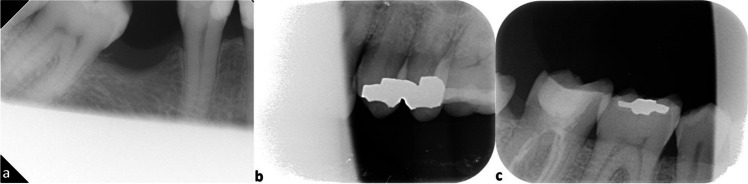
Superior premolars and molars and inferior premolars and molars radiographs with cone cutting that affects the diagnostic quality (score 2). The apex was not visible due to cone cut (**a**). The entire crown was missing due to cone cut (**b**). The bone level next to the teeth was not visible due to cone cut (**c**)

The influence on the diagnostic value of FT, SPM, and IPM was based on the following criteria:

1. The apex was not visible due to cone cut (Fig. 4a).
2. The entire crown was missing due to cone cut (Fig. 4b).
3. The bone level next to the teeth was not visible due to cone cut (Fig. 4c).

If a radiograph met one of the criteria above, it was classified as unusable and assigned a score of 2.

**Fig. 5:**
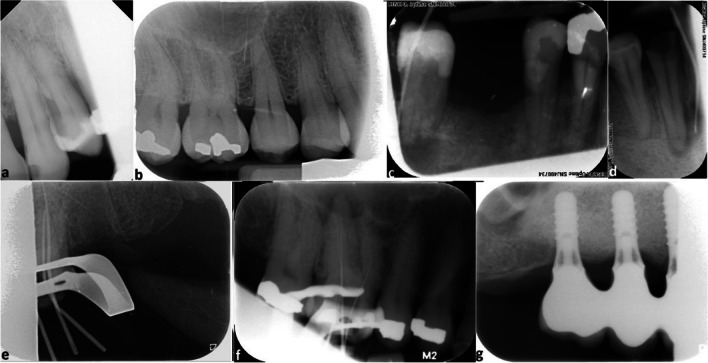
Radiographs of front teeth, superior premolars and molars, and inferior premolars and molars for which an exception was made. **a** On anterior radiographs, premolar and molar criteria did not apply. **b** For the classification of IPM and SPM into score 1 and score 2, only premolars and molars were assessed, and on an IPM or SPM radiograph, the criteria did not apply for canines or incisors. **c** When a radiograph was made for fistula tracing purposes, the end of the gutta percha point had to be visible. **d** When a radiograph was made for fistula tracing purposes, the focus was given to the tooth/teeth where the gutta percha point ended. The entire tooth with the bone level and the periapical area had to be visible. **e** When an endodontic treatment was started, the entire tooth with the bone level and the periapical area had to be visible. **f** If a radiograph was taken for length determination of the root canals, all silicon stops had to be visible. **g** If the radiograph included an implant, the entire implant with the bone level and the periapical area had to be visible

During the classification of anterior teeth into score 1 and score 2, only canine to canine was included. On an anterior radiograph, the above-mentioned criteria did not apply to premolars and molars (Fig. 5a). For the classification of IPM and SPM into score 1 and score 2, only premolars and molars were assessed, and on an IPM or SPM radiograph, the above-mentioned criteria did not apply for canines or incisors (Fig. 5b). When a radiograph was made for fistula tracing purposes, the end of the gutta percha point had to be visible. When it was missing due to a cone cut error, the radiograph was assigned a score of 2 (Fig. 5c). When a radiograph was made for fistula tracing purposes, the focus was given to the tooth/teeth where the gutta percha point ended. The entire tooth with the bone level and the periapical area had to be visible. When one of these was missing the radiograph was assigned a score of 2 (Fig. 5d). When an endodontic treatment was started, the entire tooth with the bone level and the periapical area had to be visible. If one of these was missing, the radiograph was assigned a score of 2 (Fig. 5e). If a radiograph was taken for length determination of the root canals, all silicon stops had to be visible. If one or more were missing, the radiograph was assigned a score of 2 (Fig. 5f). If the radiograph included an implant, the entire implant with the bone level and the periapical area had to be visible. If one of these was missing, the radiograph was assigned a score of 2 (Fig. 5g).

**Fig. 6:**
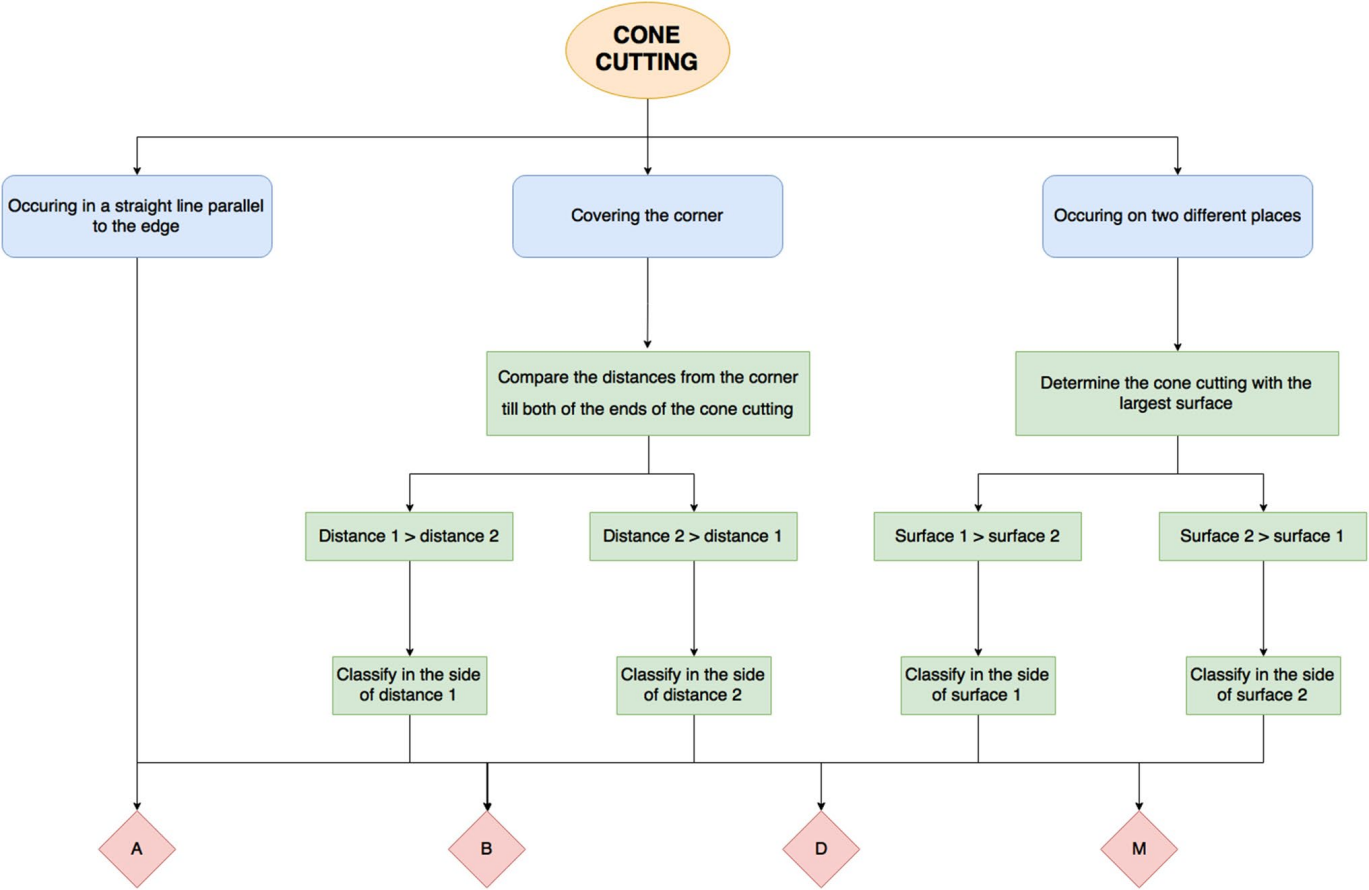
Flow chart of how the location criteria for each radiograph was applied. Location of the cone cut classified as above (A); below (B); distal (D); mesial (M)

**Fig. 7:**
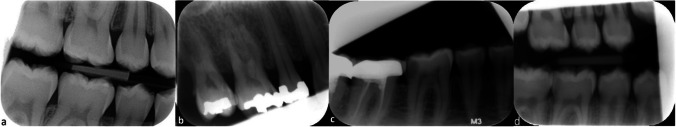
Radiographs which are divided in a group by applying the location criteria: **a** divided in group D (distal); **b** divided in group B (below); **c** divided in group A (above); **d** divided in group M (mesial)

The location of the cone cut error was also assessed and divided into four groups: cranial (labelled as above (A)), caudal (labelled as below (B)), distal (D), and mesial (M) (Fig. 6). When the cone cut did not occur in a straight line parallel to the edge of the radiograph, it was assessed by the following criteria (Fig. 7):

1. If the cone cut covered the corner of the radiograph, the distance between the corner of the radiograph and the end of the cone cut at both sides was compared. The radiograph was classified based on the side with the greatest distance (Fig. 7a, b).
2. When the cone cut occurred on two different places on the radiograph, the radiograph was classified based on the side with the largest surface (Fig. 7c, d).

The results were tabled in Microsoft Excel 2010. First, the number of radiographs in each group was tabulated. Thereafter, the percent total was calculated for score 0, score 1, and score 2, and for each group (BW, FT, SPM, and IPM). The location of the cone cut error was expressed as a percent total of the radiographs with cone cutting. This calculation was also done for the different types of radiographs.

## Results

A total of 53,684 intraoral radiographs were included in this study from the ACTA database. From this total, 8999 were radiographs in the NP group (nonclinical images like extracted teeth, referral images from external practices, or images that were too vague to classify). The remaining 44,685 radiographs were divided into BW, FT, SPM, and IPM groups and categorized with and without cone cut and cone cut but diagnostically usable versus cone cut and not diagnostically usable (Table 1). Of the 44,685 radiographs, 79% did not have any cone cut, 18% had cone cut but were diagnostically usable, and 3% of radiographs were not diagnostically usable due to cone cut.

**Table 1:** The number and percentage of radiographs divided into the different scores and ratio of scores 1 and 2

	Analyses						Subanalyses				
	Total	%	Score 0	%	Score 1 + 2	%	Score 1	%	Score 2		Ratio Score 1:score 2
Total	44,685	100%	35,348	79.10%	9337	20.90%	7850	17.57%	1487	3.33	5.28:1
BW	17,839	100%	15,018	84.19%	2821	15.81%	2332	13.07%	489	2.74	4.77:1
FT	9111	100%	7220	79.24%	1891	20.76%	1636	17.96%	255	2.80	6.42:1
SPM	9324	100%	6901	74.01%	2423	25.99%	2018	21.64%	405	4.34	4.98:1
IPM	8411	100%	6209	73.82%	2202	26.18%	1864	22.16%	338	4.02	5.51:1

Radiograph type classified as follows: bitewing (BW), front teeth (FT), superior premolar and molar (SPM), or inferior premolar and molar (IPM). The score of the radiograph: score 0: no cone cutting; score 1: cone cutting that does not affect diagnostic value; score 2: cone cutting that does affect diagnostic value

Comparing anterior periapical radiographs (group FT) with lateral periapical radiographs (groups SPM and IPM), there was less cone cutting in the anterior periapical group versus the lateral periapical groups. Periapical radiographs of the lateral area showed the most cone cut errors (26%). In the BW radiographs, cone cut was noted 16% of the time, which was the least frequent compared to all other radiographs. Even though the BW group had the lowest frequency of cone cut, the percent of radiographs that were diagnostically unusable due to cone cut was similar, ranging from 2.7% for BW, 2.8% for FT, 4.3% SPM, and 4.0% for IPM. Furthermore, radiographs with a cone cut from the FT group were less likely to be assigned a score of 2 than radiographs from the BW group (as demonstrated by the ratios in Table 1).

Out of the 9337 radiographs that had cone cut errors, 22% were classified as above, 31% below, 21% distal, and 27% mesial (Table 2). Specific to BWs, cone cut errors appeared least on the above side (16%) and almost two times as much on the mesial side (34%). In the FT group, the frequency of cone cut on the below aspect was 42%, which was almost four times as much as the cone cut on the mesial side (12%). In the SPM group, the cone cut on the above side (9%) was quite low compared to the cone cut that occurred on the below side (39%) and the mesial side (35%). Moreover, it is notable that the percentage of mesial cone cut was high in the BW and SPM groups and low in the FT group. In the IPM group, the cone cut that occurred on the above side (37%) was almost twice as much as the cone cut that occurred on the other sides (21%, 20%, and 21% respectively).

**Table 2:** The number and percentage of radiographs with cone cutting divided into the different locations

	Score 1 + 2									
	Total	%	A	%	B	%	D	%	M	%
Total	9337	100%	2048	21.93%	2887	30.92%	1918	20.54%	2484	26.60%
BW	2821	100%	462	16.38%	710	25.17%	700	24.81%	949	33.64%
FT	1891	100%	543	28.71%	791	41.83%	325	17.19%	232	12.27%
SPM	2423	100%	221	9.12%	920	37.97%	445	18.37%	837	34.54%
IPM	2202	100%	822	37.33%	466	21.16%	448	20.35%	466	21.16%

^*^A above, *B* below, *D*, distal, *M* mesial

## Discussion

This study assessed the number of cone cut errors of all radiographs taken in 2015 at ACTA and explored whether the cone cut influenced the diagnostic value of the radiographs and where at the radiograph the cone cut was most often found.

A study conducted by Shannon et al. [5] reported that the use of round collimation exposes the patient’s face to an area of 5.94 square inches of radiation. The area of radiation exposure to the patient’s face from one radiograph and one retake from a rectangular collimation is 4.82 square inches. [5] Thus, the patient is still spared 1.12 square of radiation if one retake is required using the rectangular collimator. Furthermore, the change to rectangular collimation leads to a decrease between 40 and 80% of the effective dose to the patient compared to round collimation, because of the smaller field size [2, 8]. It is worth noting that dose reduction can also be attributed to the type of sensor being used (PSP vs digital sensor) as this changes the effective dose received by the patient [8].

When using rectangular collimation and a retake is necessary, the patient would not even receive the same amount of ionizing radiation as by taking the radiograph with a round collimation in the first place. Within the present study, if 96% of the radiographs, from which the diagnostic value was not affected by cone cut, were made with a round collimator, every patient would have received approximately double the amount of ionizing radiation [8]. Similar results were found in a previous study which examined cone cuts in 6763 radiographs taken by junior dental students and found the frequency of cone cut error leading to a retake to be 2.7% [9]. Furthermore, similar to our study, the cone cut was noted to be the highest in the IPM region [9]. In summary, the rectangular collimator is effective in reducing radiation exposure to a patient.

Radiographs are frequently taken in the dental office, and as a result, the head and neck are exposed to higher doses of radiation over a patient’s lifetime. This may have the potential to induce salivary gland cancer [10], thyroid cancer [11, 12], and intracranial meningioma [12, 13]. However, it is important to note that studies associating cancer with dental radiographs were done when radiation exposure was higher with older technology [14]. Regardless of new and improved technology, dental practitioners still must follow the optimization principle [1]. Based on the findings of this study, a rectangular collimator greatly reduces the amount of radiation a patient receives without interfering with the diagnostic value of the radiograph and therefore is a textbook way for dentists to adhere to the optimization principle. Furthermore, if students can be trained to successfully take quality PSP images with the rectangular collimator, as reported by Freyche Vazquez [15] in 2023, clinicians would also likely be able to be trained to take quality images while reducing radiation to the patient with collimation.

Limitations of this study include the use of four different types of X-ray machines. The brands were not subanalyzed as it was not possible to determine which radiographs came from which machine. The radiographs were also taken by dental and dental hygiene students which may influence the study results as on one hand every student underwent training with the rectangular collimator, and on the other hand, students are less experienced in taking radiographs than dentists. Thus, we are unable to determine if the partial exposures are exclusively related to the rectangular collimator or the operator experience level, and as such, if the results would be different if round collimation was used. Furthermore, the criteria for score 1 and score 2 were different for the BW group versus the FT, SPM, and IPM groups. The results showed a lower percentage of cone cut in the BW group compared to the other groups and this may have been influenced by the different criteria. This was because the periapical radiographs had more specific criteria, and if one area was missed, the radiograph was immediately assigned a score of 2. Future directions include a prospective study including why the radiographs were prescribed. This data would help inform if the diagnostic value was affected due to cone cutting as the present data is unable to determine if a retake was truly necessary. For a prospective or multiple-center study, it is worth considering the level of education and use of the same type of radiographic machine, standardizing the characteristics of each radiographic machine such as stability and number of unit arms, and comparing rectangular and round collimation for the same group of professionals. Future research exploring the cause of the cone cut errors may also be useful. For example, whether the collimator was misaligned, or if the patient moved or swallowed.

Alongside these results to support the use of a rectangular collimator is the dental care professional’s obligation to adhere to the optimization principle [1] and consequently practice radiation stewardship. Radiation stewardship is analogues to antibiotic stewardship which is the movement to measure and improve antibiotic prescriptions to reduce the negative outcomes of unnecessary or improper use (CDC) [16]. Antibiotic stewardship influences recommendations from (inter)national guidelines and therefore clinical decisions in daily practice. Radiation stewardship is the implementation of the optimization principle. In medical and dental radiography, this principle was further detailed by the introduction of the acronym ALADA in 2017 [17]. The term ALADA stands for “as low as diagnostically acceptable” and is sometimes extended to “as low as diagnostically acceptable being indication-oriented and patient-specific” (ALADAIP) [17]. The purpose of this term is to emphasize the need to optimize radiation rather than simply minimize it. Several strategies exist to minimize radiation exposure to the patient including beam collimation [18]. Thus, even though new radiation technology limits the overall radiation a patient receives, proper, individualized prescription of radiographs remains necessary [18]. By practicing according to these principles, the dental professional practices an important point of radiation stewardship.

## Conclusion

The frequency of cone cut errors interfering with diagnostics from using a rectangular collimator in intraoral radiography is very limited, and thus, applying the ALADA principle and radiation stewardship, the dental practitioner should choose rectangular collimation instead of round collimation.

## Data Availability

The data that support the findings of this study are available from the corresponding author upon reasonable request.

## Appendix

**Table:**

	Item no	STROBE items	Location in manuscript where items are reported	RECORD items	Location in manuscript where items are reported
Title and abstract					
	1	(a) Indicate the study’s design with a commonly used term in the title or the abstract. (b) Provide in the abstract an informative and balanced summary of what was done and what was found	Pages 1 and 3	RECORD 1.1: the type of data used should be specified in the title or abstract. When possible, the name of the databases used should be included RECORD 1.2: if applicable, the geographic region and timeframe within which the study took place should be reported in the title or abstract RECORD 1.3: if linkage between databases was conducted for the study, this should be clearly stated in the title or abstract	Page 3 Page 3 n/a
Introduction					
Background rationale	2	Explain the scientific background and rationale for the investigation being reported	Page 4/5		
Objectives	3	State-specific objectives, including any prespecified hypotheses	Page 3/5		
Methods					
Study design	4	Present key elements of study design early in the paper	Page 6/7		
Setting	5	Describe the setting, locations, and relevant dates, including periods of recruitment, exposure, follow-up, and data collection	Page 6/7		
Participants	6	*(a) Cohort study*—give the eligibility criteria, and the sources and methods of selection of participants. Describe methods of follow-up *Case–control study*—give the eligibility criteria, and the sources and methods of case ascertainment and control selection. Give the rationale for the choice of cases and controls *Cross-sectional study*—give the eligibility criteria, and the sources and methods of selection of participants *(b) Cohort study*—for matched studies, give matching criteria and number of exposed and unexposed *Case–control study*—for matched studies, give matching criteria and the number of controls per case	Page 6/7 n/a n/a n/a n/a	RECORD 6.1: the methods of study population selection (such as codes or algorithms used to identify subjects) should be listed in detail. If this is not possible, an explanation should be provided RECORD 6.2: any validation studies of the codes or algorithms used to select the population should be referenced. If validation was conducted for this study and not published elsewhere, detailed methods and results should be provided RECORD 6.3: if the study involved linkage of databases, consider use of a flow diagram or other graphical display to demonstrate the data linkage process, including the number of individuals with linked data at each stage	Pages 5–7 n/a n/a
Variables	7	Clearly define all outcomes, exposures, predictors, potential confounders, and effect modifiers. Give diagnostic criteria, if applicable	Page 6/7	RECORD 7.1: a complete list of codes and algorithms used to classify exposures, outcomes, confounders, and effect modifiers should be provided. If these cannot be reported, an explanation should be provided	n/a
Data sources/measurement	8	For each variable of interest, give sources of data and details of methods of assessment (measurement) Describe comparability of assessment methods if there is more than one group	Page 6/7		
Bias	9	Describe any efforts to address potential sources of bias	Page 6		
Study size	10	Explain how the study size was arrived at	Page 6		
Quantitative variables	11	Explain how quantitative variables were handled in the analyses. If applicable, describe which groupings were chosen, and why	Page 6/7		
Statistical methods	12	(a) Describe all statistical methods, including those used to control for confounding (b) Describe any methods used to examine subgroups and interactions (c) Explain how missing data were addressed (d) *Cohort study*—if applicable, explain how loss to follow-up was addressed *Case–control study*—if applicable, explain how matching of cases and controls was addressed *Cross-sectional study*—if applicable, describe analytical methods taking account of sampling strategy (e) Describe any sensitivity analyses	Page 6/7		
Data access and cleaning methods		.		RECORD 12.1: authors should describe the extent to which the investigators had access to the database population used to create the study population RECORD 12.2: authors should provide information on the data cleaning methods used in the study	Page 6
Linkage		.		RECORD 12.3: state whether the study included person-level, institutional-level, or other data linkage across two or more databases. The methods of linkage and methods of linkage quality evaluation should be provided	n/a
Results					
Participants	13	(a) Report the numbers of individuals at each stage of the study (e.g., numbers potentially eligible, examined for eligibility, confirmed eligible, included in the study, completing follow-up, and analyzed) (b) Give reasons for non-participation at each stage (c) Consider use of a flow diagram	Page 8 Page 8 Figure 1, Table 1/2	RECORD 13.1: describe in detail the selection of the persons included in the study (i.e., study population selection) including filtering based on data quality, data availability, and linkage. The selection of included persons can be described in the text and/or by means of the study flow diagram	Page 6
Descriptive data	14	(a) Give characteristics of study participants (e.g., demographic, clinical, social) and information on exposures and potential confounders (b) Indicate the number of participants with missing data for each variable of interest (c) *Cohort study*—summarize follow-up time (e.g., average and total amount)	Page 8 n/a		
Outcome data	15	*Cohort study*—report numbers of outcome events or summary measures over time *Case–control study*—report numbers in each exposure category, or summary measures of exposure *Cross-sectional study*—report numbers of outcome events or summary measures	n/a		
Main results	16	(a) Give unadjusted estimates and, if applicable, confounder-adjusted estimates and their precision (e.g., 95% confidence interval). Make clear which confounders were adjusted for and why they were included (b) Report category boundaries when continuous variables were categorized (c) If relevant, consider translating estimates of relative risk into absolute risk for a meaningful time period	Page 8		
Other analyses	17	Report other analyses done—e.g., analyses of subgroups and interactions, and sensitivity analyses	Page 8		
Discussion					
Key results	18	Summarize key results with reference to study objectives	Page 9		
Limitations	19	Discuss limitations of the study, taking into account sources of potential bias or imprecision. Discuss both direction and magnitude of any potential bias	Page 9	RECORD 19.1: discuss the implications of using data that were not created or collected to answer the specific research question(s). Include discussion of misclassification bias, unmeasured confounding, missing data, and changing eligibility over time, as they pertain to the study being reported	Page 9
Interpretation	20	Give a cautious overall interpretation of results considering objectives, limitations, multiplicity of analyses, results from similar studies, and other relevant evidence	Page 10		
Generalisability	21	Discuss the generalisability (external validity) of the study results	Page 10		
Other information					
Funding	22	Give the source of funding and the role of the funders for the present study and, if applicable, for the original study on which the present article is based	Page 1		
Accessibility of protocol, raw data, and programming code		.	n/a	RECORD 22.1: authors should provide information on how to access any supplemental information such as the study protocol, raw data, or programming code	n/a

*Reference: Benchimol EI, Smeeth L, Guttmann A, Harron K, Moher D, Petersen I, Sørensen HT, von Elm E, Langan SM, the RECORD Working Committee. The REporting of studies Conducted using Observational Routinely collected health Data (RECORD) Statement. *PLoS Medicine* 2015; in press

*Checklist is protected under Creative Commons Attribution (CC BY) license
